# Identification and characterization of a TGF-**β**-independent SMAD4–NFATc1–STAT3 regulatory axis

**DOI:** 10.1093/jmcb/mjaf028

**Published:** 2025-08-26

**Authors:** Wukun Ouyang, Jiaying Hao, Qiankun Niu, Eugene F Douglass, Christian M Beusch, David E Gordon, Maggie Hall, Richard A Moffitt, Yuhong Du, Xiulei Mo

**Affiliations:** Department of Pharmacology and Chemical Biology, Emory University School of Medicine, Atlanta, GA 30322, USA; Department of Pharmacology and Chemical Biology, Emory University School of Medicine, Atlanta, GA 30322, USA; Department of Pharmacology and Chemical Biology, Emory University School of Medicine, Atlanta, GA 30322, USA; Department of Pharmaceutical and Biomedical Sciences, University of Georgia, Athens, GA 30602, USA; Department of Pathology and Laboratory Medicine, Emory University School of Medicine, Atlanta, GA 30322, USA; Department of Pathology and Laboratory Medicine, Emory University School of Medicine, Atlanta, GA 30322, USA; Department of Hematology and Medical Oncology, Emory University, Atlanta, GA 30322, USA; Department of Hematology and Medical Oncology, Emory University, Atlanta, GA 30322, USA; Department of Biomedical Informatics, Emory University, Atlanta, GA 30322, USA; Department of Pharmacology and Chemical Biology, Emory University School of Medicine, Atlanta, GA 30322, USA; Emory Chemical Biology Discovery Center, Emory University School of Medicine, Atlanta, GA 30322, USA; Department of Pharmacology and Chemical Biology, Emory University School of Medicine, Atlanta, GA 30322, USA

**Keywords:** SMAD4, protein–protein interaction, NFATc1, STAT3, OncoPPI

## Abstract

SMAD4, a central mediator of the TGF-**β** signaling pathway, plays a critical role in regulating cellular processes such as proliferation, differentiation, and apoptosis. While SMAD4’s canonical functions within TGF-**β** signaling are well-established, its non-canonical, TGF-**β**-independent roles remain poorly understood, particularly in the context of disease biology. Here, we investigate SMAD4’s TGF-**β**-independent functions by identifying and characterizing its protein–protein interaction network. Using pancreatic ductal adenocarcinoma as a model system, we performed a SMAD4-focused oncogenic protein–protein interaction mapping and uncovered a novel interaction between SMAD4 and NFATc1. We demonstrated that SMAD4 binds to NFATc1 in a phosphorylation-dependent but TGF-**β**-independent manner, sequestering NFATc1 in the cytoplasm and inhibiting its transcriptional activity. The absence of this interaction in SMAD4-deficient PDAC cells is associated with the activation of NFATc1 transcriptional programs and upregulation of STAT3 at both mRNA and protein levels. Pharmacological profiling revealed multiple STAT3 inhibitors with selective efficacy against SMAD4-deficient PDAC cells *in vitro*, highlighting a potential therapeutic vulnerability. These findings identify a previously uncharacterized SMAD4–NFATc1 regulatory complex and establish its biological significance in regulating NFATc1-driven transcriptional programs, such as STAT3, providing critical insights into SMAD4’s TGF-**β**-independent functions and uncovering new opportunities for therapeutic intervention in SMAD4-deficient contexts.

## Introduction

SMAD4, a transcriptional master regulator in the TGF-**β** signaling pathway, is essential for critical cellular processes, such as differentiation, growth, and apoptosis (Zhao et al., 2018). SMAD4 performs its canonical adaptor function by forming protein–protein interactions (PPIs) with receptor-regulated SMADs (r-SMADs), such as SMAD2 and SMAD3 (Ouyang et al., 2023), facilitating the transduction of TGF-**β** signaling from the cytoplasm to the nucleus. While this canonical role of SMAD4 is well-established, emerging evidence suggests that SMAD4 also exhibits TGF-**β**-independent functions in diverse biological contexts, such as cancer (Mo et al., 2022), infectious diseases (Gowripalan et al., 2020), and immune responses (Wang et al., 2018b; Igalouzene et al., 2022; Chandiran and Cauley, 2023). However, the mechanisms underlying these non-canonical roles remain poorly understood (Lindsay et al., 2025). Elucidating the SMAD4 PPI network could provide a deeper understanding of these TGF-**β**-independent functions and reveal novel therapeutic opportunities in disease contexts.

Cancer genomic studies have identified the *SMAD4* gene as one of the most frequently mutated genes across multiple malignancies, including pancreatic, colon, and lung cancers (Shugang et al., 2016; Zhao et al., 2018; Song et al., 2023). In pancreatic ductal adenocarcinoma (PDAC), *SMAD4* mutations are particularly prevalent, occurring in ∼50% of cases, and are strongly associated with shorter survival and poor prognosis. PDAC remains one of the most challenging malignancies to treat, largely due to its resistance to existing therapeutic modalities (Kleeff et al., 2016; Park et al., 2021). Its lethality stems from multifaceted challenges, including frequent late-stage diagnosis, which limits surgical intervention, and a dense stromal tumor microenvironment that impedes drug delivery and reduces the efficacy of standard chemotherapies (Hosein et al., 2020). Intratumoral genetic heterogeneity further complicates treatment by hindering the development of universally applicable targeted therapies (Gutierrez et al., 2021). Moreover, while immunotherapies have shown remarkable success in other cancers, their utility in PDAC remains limited (Timmer et al., 2021). Collectively, these therapeutic challenges underscore the urgent need for innovative treatment approaches and a deeper understanding of PDAC biology.

Genomic analyses have identified recurrent somatic alterations in key PDAC driver genes, including *KRAS, CDKN2A, TP53*, and *SMAD4* (Hayashi et al., 2021). While considerable efforts have been made to develop therapeutic strategies targeting these genes, they remain largely undrugged (Tang et al., 2021; Cox and Der, 2024). Among these drivers, SMAD4 mutations are particularly significant due to their role in disrupting normal cell cycle regulation, promoting tumorigenesis and metastasis, and contributing to therapy resistance (Shugang et al., 2016; Wang et al., 2018a; Principe et al., 2022; Song et al., 2023). Unlike gain-of-function oncogene mutations that offer clear therapeutic targets, SMAD4 tumor suppressor gene mutations typically result in loss-of-function variants, presenting substantial challenges for therapeutic intervention. These factors make understanding SMAD4-associated pathways an urgent priority in the quest for effective PDAC treatments.

To address these gaps, we conducted an oncogenic PPI (OncoPPI) profiling to systematically identify previously unreported physical interactions between SMAD4 and cancer-associated proteins (Li et al., 2017). By employing a multidisciplinary approach that integrates systems biology, bioinformatics, biochemical assays, genetic manipulation, and pharmacological perturbation, we investigated how SMAD4 loss reshapes oncogenic signaling using PDAC models. Our study uncovered a rewired SMAD4–NFATc1–STAT3 signaling axis and demonstrated a collateral therapeutic vulnerability of SMAD4-deficient PDAC cells to STAT3 inhibitors. These findings not only provide new insights into SMAD4’s TGF-**β**-independent functions but also reveal actionable vulnerabilities that can inform precision medicine strategies for PDAC.

## Results

### OncoPPI screening identifies SMAD4 protein partners

PDAC characterized by SMAD4 loss represents a distinct genetic subtype associated with poor prognosis and resistance to therapy (Fei et al., 2021; Principe et al., 2022). Despite the critical role of SMAD4 in tumor suppression, the molecular mechanisms linking SMAD4 loss to oncogenesis remain poorly understood and cannot be solely attributed to disruptions in canonical TGF-**β** signaling. To address this gap, we conducted OncoPPI mapping to systematically identify SMAD4-binding partners and explore their contributions to oncogenic signaling.

**Figure 1:**
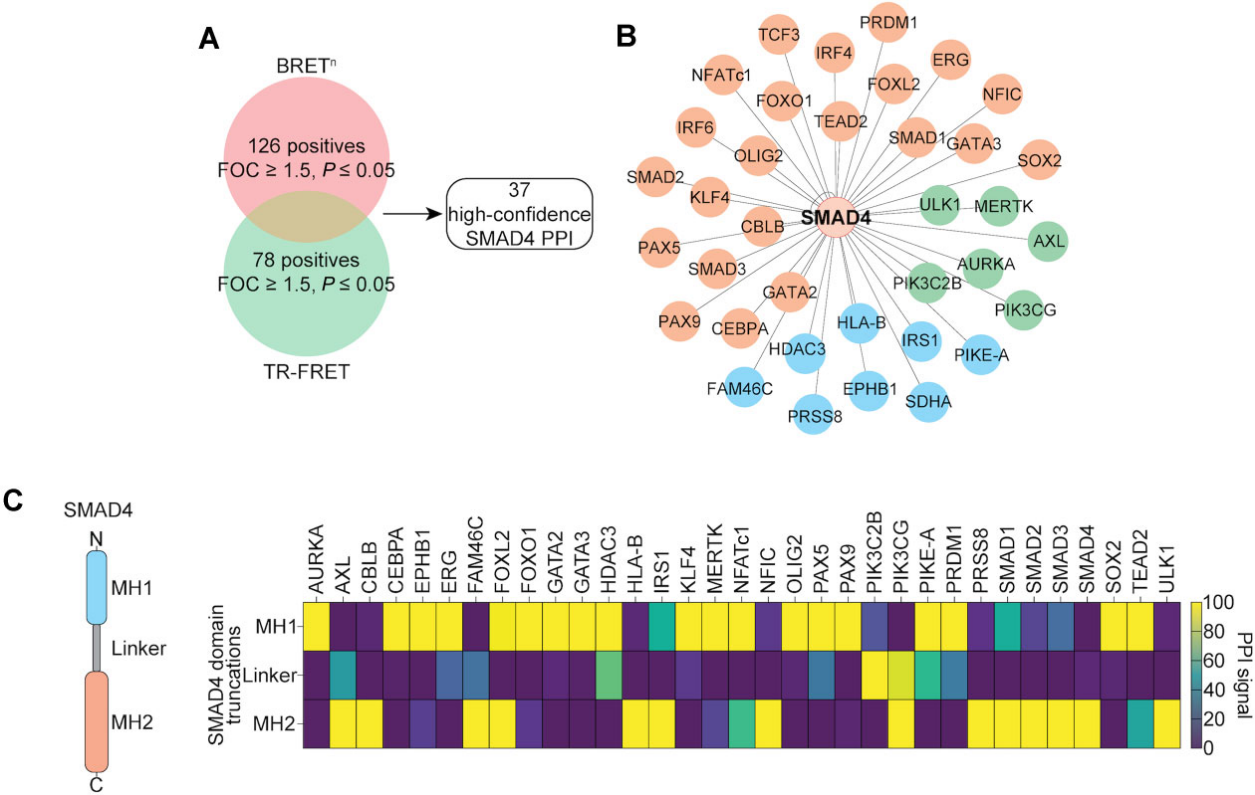
OncoPPI profiling identifies the SMAD4 PPI hub. (**A**) Venn diagram illustrating positive PPIs identified from BRET^n^- and TR-FRET-based OncoPPI screening. PPIs were prioritized using a cutoff of FOC ≥ 1.5 and *P* ≤ 0.05. (**B**) Spoke diagram depicting the high-confidence SMAD4 OncoPPI hub. Binding partners are categorized into transcription factors (orange), kinases (green), and other regulatory proteins (blue). (**C**) Schematic representation of SMAD4 domains (left) and heatmap of TR-FRET PPI signals (right) for SMAD4 domain truncations and their corresponding binding partners. PPI signals for each binding partner are normalized to the smallest and largest values in the dataset.

Using our OncoPPI v2 gene library (Mo et al., 2022), which comprises 556 wild-type (WT) human protein-coding genes, we screened for SMAD4 interactions with oncogenic drivers and regulatory proteins. The initial screen employed bioluminescence resonance energy transfer (BRET^n^) technology in live cells (Mo et al., 2022), enabling the detection of direct interactions between Nanoluc (Nluc)-tagged SMAD4 and Venus-tagged OncoPPI proteins. This approach identified 126 positive hits based on a stringent fold-of-change (FOC) ≥ 1.5 and *P* ≤ 0.05 (Figure 1A). To confirm these interactions, we performed an orthogonal screen using time-resolved fluorescence resonance energy transfer (TR-FRET) in cell lysates (Li et al., 2017), yielding 78 positive hits. Integrating these results, we prioritized 37 high-confidence SMAD4 OncoPPIs (Figure 1A).

The identified SMAD4 OncoPPIs comprise 22 transcription factors, 6 kinases, and 9 additional regulatory proteins (Figure 1B). This dataset rediscovered established SMAD4 partners, including SMAD1/2/3 (Nakao et al., 1997; Ouyang et al., 2023), GATA2 (Dong et al., 2014), and FOXO1 (Seoane et al., 2004), while also revealing previously unreported interactors such as NFATc1 (Figure 1B; Macian, 2005). Validation using affinity-based glutathione S-transferase (GST)-pulldown assays confirmed robust interactions for 33 OncoPPIs, including significant enrichment of Venus-Flag-tagged NFATc1 (VF-NFATc1) in complex with GST-SMAD4 but not with the negative controls (Figure 1B).

SMAD4’s multi-domain architecture (de Caestecker et al., 1997), which includes an MH1 DNA-binding domain (DBD), a flexible linker region, and an MH2 protein interaction domain, suggests diverse interaction modalities (Figure 1C). To map these interactions, we performed TR-FRET-based domain truncation studies. Binding signals revealed three distinct interaction classes: the MH2 domain interacted strongly with SMAD1/2/3, consistent with its known role in canonical TGF-**β** signaling (Nakao et al., 1997); the MH1 domain preferentially bound to transcription factors such as FOXO1, GATA2, and NFATc1; and the linker region demonstrated selective interactions with proteins like PIK3C2B (Figure 1C). These findings highlight the versatility of SMAD4 as a regulatory hub and suggest potential pathway rewiring in SMAD4-mutant PDAC.

### Identification of NFATc1 as a critical regulator in SMAD4-deficient PDAC cells

**Figure 2:**
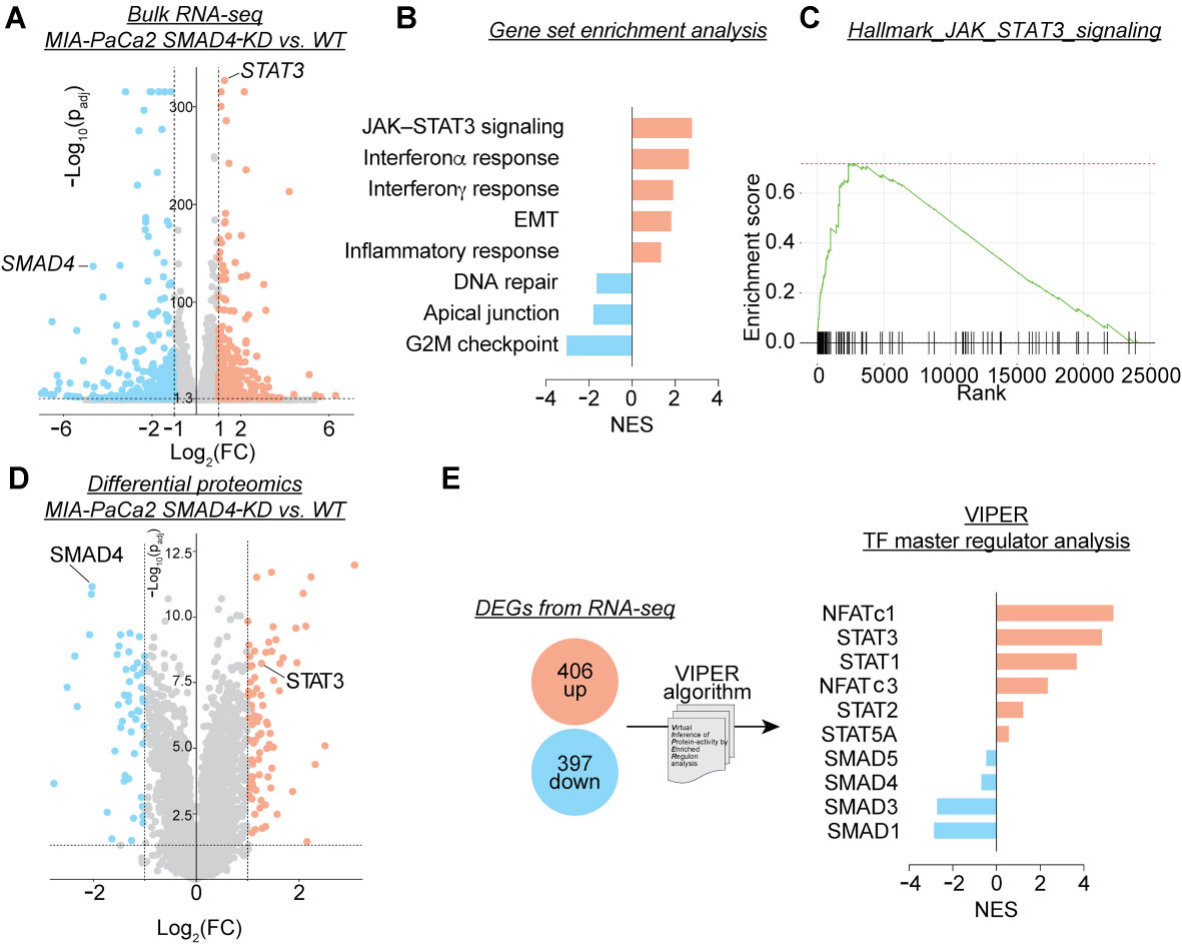
Identification of NFATc1 as a reprogrammed transcriptional master regulator in SMAD4-KD PDAC cells. (**A**) Volcano plot showing DEGs in SMAD4-KD MIA-PaCa2 cells compared to WT controls. (**B**) Significantly altered pathways associated with DEGs. (**C**) Upregulation of JAK–STAT3 signaling in SMAD4-KD MIA-PaCa2 cells. (**D**) Volcano plot depicting differentially expressed proteins in SMAD4-KD MIA-PaCa2 cells compared to WT controls. (**E**) VIPER analysis identifying significantly altered transcriptional master regulators based on DEGs from transcriptomic profiling.

To further explore the functional relevance of SMAD4 interactions, we focused on identifying master regulators implicated in SMAD4 mutation-driven rewiring of oncogenic pathways in PDAC. We generated isogenic SMAD4-knockdown (KD) Mia-Paca2 cell lines to examine transcriptomic and proteomic changes associated with SMAD4 loss. Transcriptomic profiling identified 406 upregulated and 397 downregulated genes in SMAD4-KD cells, reflecting extensive transcriptional rewiring (Figure 2A). Gene set enrichment analysis (GSEA) revealed alterations in both canonical and non-canonical SMAD4-associated pathways. Canonical pathways included the downregulation of G2/M checkpoint regulation and upregulation of epithelial-to-mesenchymal transition (EMT), while non-canonical pathways featured significant downregulation of DNA repair processes and activation of JAK–STAT3 signaling (Figure 2B and C).

Proteomic profiling further validated these findings, identifying 88 upregulated and 51 downregulated proteins in SMAD4-KD cells (Figure 2D). Both differential transcriptome and proteome datasets confirmed efficient SMAD4 KD, as evidenced by reduced SMAD4 expression at mRNA and protein levels. Notably, STAT3 was consistently upregulated in SMAD4-KD cells, aligning with the observed activation of JAK–STAT3 signaling (Figure 2C).

To identify upstream transcriptional regulators driving these changes, we conducted virtual inference of protein activity by enriched regulon (VIPER) (Alvarez et al., 2016) analysis on the differentially expressed genes (DEGs). Consistent with SMAD4 loss, transcriptional activities of r-SMADs, such as SMAD1 and SMAD3, were reduced (Figure 2E). Concurrently, STAT3 activity was significantly elevated, corroborating its upregulation at transcript and protein levels (Figure 2A–D). Interestingly, NFATc1 emerged as one of the top-ranked upregulated master regulators associated with SMAD4 KD-induced DEGs (Figure 2D), suggesting a pivotal role for NFATc1 in orchestrating transcriptional rewiring in SMAD4-deficient PDAC.

### SMAD4 physically interacts with NFATc1 in PDAC cells

Findings from both OncoPPI mapping and transcriptomic profiling identified NFATc1 as a potential SMAD4-binding partner (Figure 1) and a reprogrammed transcriptional master regulator in SMAD4-KD PDAC cells (Figure 2). Further survival analysis of the TCGA-PDAC cohort dataset revealed that SMAD4 mutant plus NFATc1 overexpression predicts a significantly worse survival (Supplementary Figure S1A). Such convergence of physical interaction and functional correlation evidence suggests the presence of a rewired SMAD4–NFATc1 OncoPPI in SMAD4-mutant PDAC, warranting further validation and characterization.

**Figure 3:**
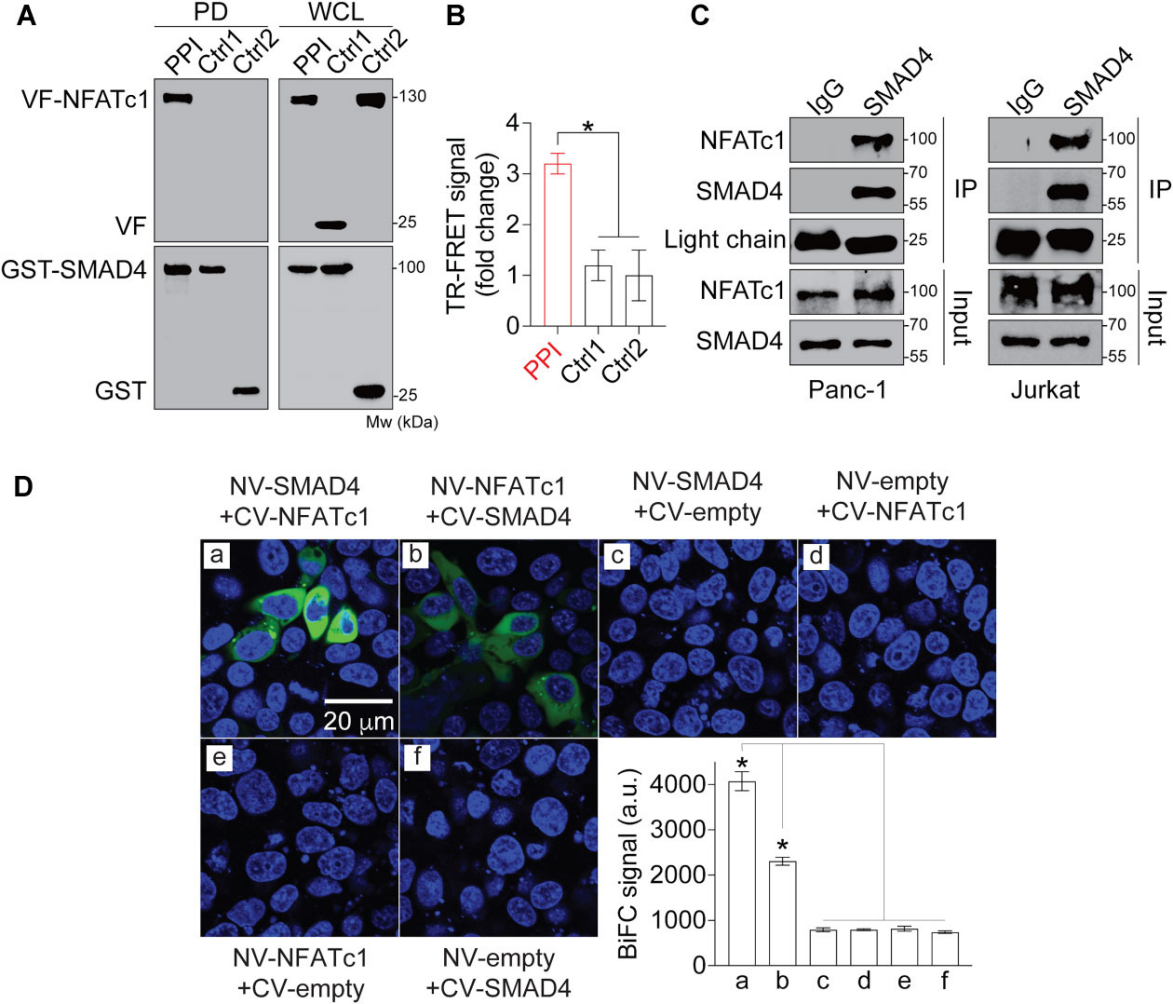
Validation of the SMAD4–NFATc1 PPI. (**A**) GST-pulldown confirmation of the SMAD4–NFATc1 PPI. HEK293T cell lysates co-expressing GST-SMAD4 with VF-NFATc1 (PPI), GST-SMAD4 with VF-empty (Ctrl1), or GST-empty with VF-NFATc1 (Ctrl2) were subjected to pulldown (PD) followed by immunoblotting. WCL, whole-cell lysate. (**B**) TR-FRET PPI signal between GST-SMAD4 and VF-NFATc1 or corresponding controls. PPI signals are expressed as fold change over the lowest empty vector control (Ctrl2) and presented as mean ± SD from three independent experiments. (**C**) Endogenous co-IP demonstrating the SMAD4–NFATc1 PPI in Panc-1 and Jurkat cells. (**D**) Cytoplasmic localization of the SMAD4–NFATc1 PPI in Panc-1 cells transfected with N-Venus-tagged SMAD4 (NV-SMAD4) and C-Venus-tagged NFATc1 (CV-NFATc1) (panel a) or *vice versa* (panel b), with corresponding controls (panels c–f). Green indicates reconstituted Venus fluorescence; blue indicates nuclear staining with Hoechst. BiFC signals were measured in cell lysates and presented as mean ± SD of fluorescence intensity from three independent experiments. *P ≤ 0.05 was calculated using the unpaired two-tailed Student's *t*-test without adjustments compared to controls.

To confirm the physical interaction between SMAD4 and NFATc1, we employed multiple orthogonal PPI detection assays. In the affinity-based GST-pulldown assay, we observed that VF-NFATc1 co-precipitated with GST-SMAD4, while no interaction was detected with the GST-empty or VF-empty negative controls (Figure 3A). These results demonstrated a strong affinity between SMAD4 and NFATc1. Using the proximity-based TR-FRET assay, we detected a significant PPI signal (FOC > 3, *P* < 0.05) between GST-SMAD4 and VF-NFATc1, compared to negative controls, indicating a direct physical interaction (Figure 3B).

To validate the SMAD4–NFATc1 interaction under more physiologically relevant conditions with endogenous protein expression, we performed a co-immunoprecipitation (co-IP) assay. In Panc-1 cells, a PDAC cell line expressing WT SMAD4, NFATc1 was consistently co-precipitated with SMAD4, whereas no such interaction was observed with the IgG negative control (Figure 3C). The SMAD4–NFATc1 PPI was also validated in Jurkat T, MIA-PaCa-2, and Capan-2 PDAC cells at the endogenous level (Figure 3C; Supplementary Figure S1B). Furthermore, we performed the bimolecular fluorescence complementation assay (BiFC) to visualize the cellular localization of the SMAD4–NFATc1 complex. Panc-1 cells co-expressing SMAD4 and NFATc1 BiFC constructs displayed strong Venus fluorescence signals in the cytoplasm, while empty vector controls showed undetectable fluorescence (Figure 3D). These findings suggest that the SMAD4–NFATc1 interaction primarily occurs in the cytoplasm.

Taken together, these orthogonal PPI assays provide robust evidence for the physical interaction between SMAD4 and NFATc1 in PDAC cells, establishing a foundation for further investigation into their functional relevance.

### SMAD4–NFATc1 PPI is mediated by SMAD4 and NFATc1 DNA-binding domains

**Figure 4:**
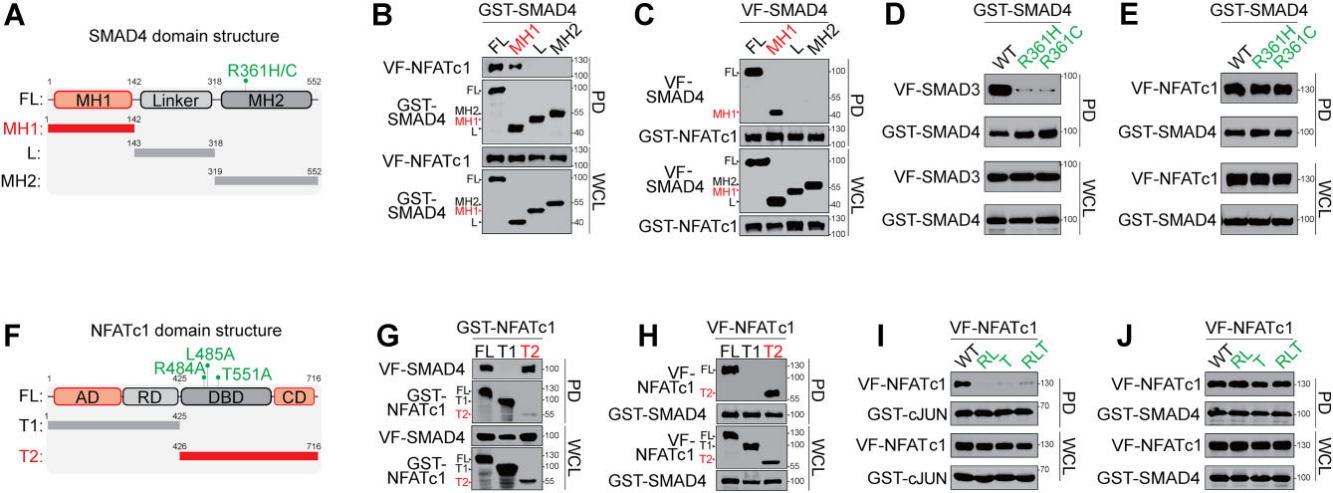
Mapping of the SMAD4–NFATc1 PPI interface. (**A**) SMAD4 domain structures and cancer-associated mutations. FL, full-length; MH1, MH1 domain truncation (1–142 amino acids); L, linker region truncation (143–318 amino acids); MH2, MH2 domain truncation (319–552 amino acids). (**B** and **C**) Mapping the NFATc1-binding domain on SMAD4 by GST-pulldown analysis. HEK293T cells were co-transfected with VF-NFATc1 and GST-tagged SMAD4 FL, MH1, L, or MH2 (**B**) or with GST-NFATc1 and VF-tagged SMAD4 FL or truncations (**C**). (**D** and **E**) Cancer-associated loss-of-function SMAD4 variants (R361H and R361C), which are deficient in binding to SMAD3 (**D**), retain the interaction with NFATc1 (**E**). HEK293T cells were co-transfected with VF-tagged SMAD3 or NFATc1 and GST-tagged SMAD4 WT or mutants, followed by GST-pulldown analysis. (**F**) NFATc1 domain structures and mutations. T1, truncation of AD and RD (1–425 amino acids); T2, truncation of DBD and CD (426–716 amino acids). (**G** and **H**) Mapping the SMAD4-binding domain on NFATc1 by GST-pulldown analysis. HEK293T cells were co-transfected with VF-SMAD4 and GST-tagged NFATc1 FL, T1, or T2 (**G**) or with GST-SMAD4 and VF-tagged NFATc1 FL or truncations (**H**). (**I** and **J**) NFATc1 mutants deficient in binding to cJUN (**I**) retain the interaction with SMAD4 (**J**). HEK293T cells were co-transfected with GST-tagged cJUN or SMAD4 and VF-tagged NFATc1 WT or loss-of-function mutants (R484A/L485A, T551A, and R484A/L485A/T551A), followed by GST-pulldown analysis. Data are presented using representative images from three independent experiments.

To further characterize the structural basis of the SMAD4–NFATc1 interaction, we performed protein domain truncation studies. We first generated truncations of SMAD4, including the MH1 DBD, the flexible linker region (L), and the MH2 protein interaction domain, respectively (Figure 4A). GST-pulldown assays revealed that NFATc1 interacted robustly with full-length SMAD4 (FL) and the MH1 domain, but not with the linker or MH2 domain (Figure 4B and C). These findings corroborated the results from TR-FRET domain profiling (Figure 1C), establishing that the SMAD4–NFATc1 PPI is predominantly mediated by the MH1 domain of SMAD4.

To further validate this binding mode, we tested the effects of two SMAD4 mutations, R361H and R361C, associated with PDAC (Tang et al., 2021; Mo et al., 2022; Ouyang et al., 2023), on SMAD4 interactions. These mutations, located in the MH2 domain, disrupted the SMAD4–SMAD3 interaction (Figure 4D), consistent with the known structural basis of the SMAD4–SMAD3 PPI. In contrast, the SMAD4 R361H and R361C mutants retained their interaction with VF-NFATc1 at levels comparable to WT SMAD4 (Figure 4D). To further assess the role of the MH1 domain, we examined two naturally occurring SMAD4 truncation mutants, G89* and G145*, which retain partial MH1 domain. Both mutants showed significantly reduced SMAD4–NFATc1 PPI in the TR-FRET assay (Supplementary Figure S1C). These results suggest that the SMAD4–NFATc1 interaction is dependent on the MH1 domain and independent of SMAD3 binding, supporting a mechanism distinct from canonical TGF-**β** signaling.

Reciprocally, we examined the structural basis of NFATc1 in mediating its interaction with SMAD4. Truncation studies divided NFATc1 into two regions: the N-terminal T1 truncation, containing the activation domain (AD) and regulatory domain (RD), and the C-terminal T2 truncation, encompassing the DBD and C-terminal domain (CD). GST-pulldown assays showed that SMAD4 interacted with full-length NFATc1 (FL) and the T2 truncation, but not with the T1 truncation (Figure 4G and H). These results indicate that the SMAD4–NFATc1 PPI is mediated primarily by the C-terminal DBD and CD regions of NFATc1.

Given that NFATc1 interacts with cJUN (Jain et al., 1993), a canonical binding partner, through its DBD, we investigated whether SMAD4 engages NFATc1 via the same interface. We tested the impact of NFATc1 mutations (R484A, L485A, and T551A), known to disrupt cJUN binding (Macian et al., 2000), on NFATc1 interactions. As expected, T551A (T), R484A/L485A (RL), and R484A/L485A/T551A (RLT) all significantly impaired cJUN binding (Figure 4I), confirming the critical role of these residues in the NFATc1–cJUN PPI (Chen et al., 1998). However, the same mutants maintained their interaction with SMAD4 at levels comparable to WT NFATc1 (Figure 4J). These findings demonstrate that the SMAD4–NFATc1 interaction is mediated by a distinct interface involving the DBD and CD regions of NFATc1, independent of its binding to cJUN.

### SMAD4–NFATc1 PPI inhibits NFATc1 transcriptional activity

**Figure 5:**
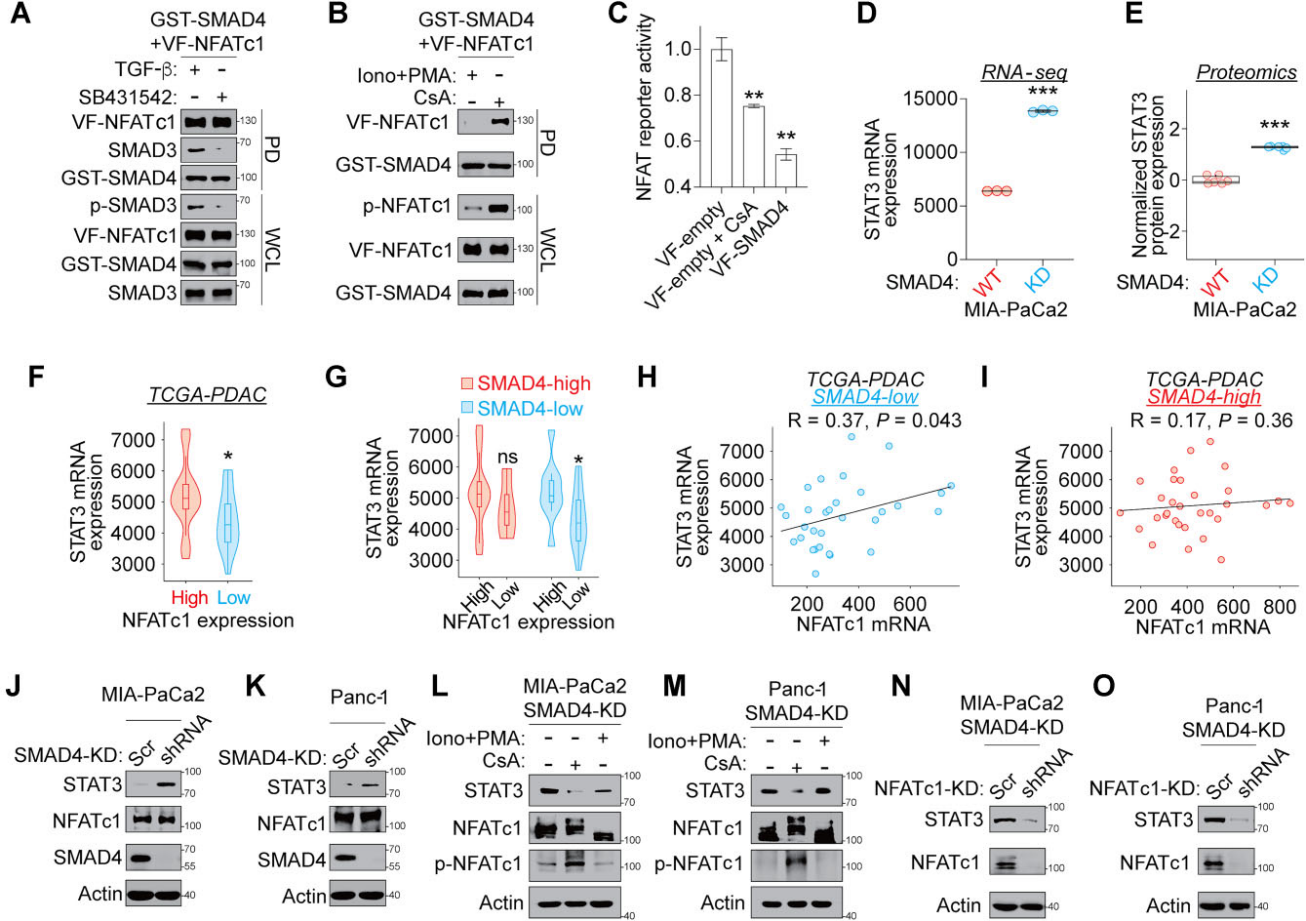
Identification of the SMAD4–NFATc1–STAT3 regulatory axis in PDAC cells. (**A**) The SMAD4–NFATc1 PPI is independent of canonical TGF-**β** signaling. HEK293T cells expressing GST-SMAD4 and VF-NFATc1 were treated with human recombinant TGF-**β** (10 ng/ml, 4 h) or the TGF-**β** receptor inhibitor SB431542 (10 μM, 1 h). (**B**) The phosphorylation dependency of the SMAD4–NFATc1 PPI. HEK293T cells expressing GST-SMAD4 and VF-NFATc1 were treated with 10 μM ionomycin and 1 μM PMA (Iono+PMA, 4 h) or CsA (10 μM, 1 h). (**C**) NFATc1 luciferase reporter activity in HEK293T cells expressing VF-empty or VF-SMAD4 and treated with CsA (10 μM, 4 h). Data are normalized to the VF-empty control and presented as mean ± SD from three independent experiments. **P ≤ 0.01 from student's *t*-test. (**D** and **E**) Box plots showing STAT3 mRNA (**D**) and protein (**E**) expression levels in isogenic SMAD4-WT and SMAD4-KD MIA-PaCa2 cells. Data represent mean ± SD from three technical replicates. ***P ≤ 0.001 from student's *t*-test. (**F**–**I**) STAT3 mRNA expression in TCGA-PDAC patient samples was grouped by NFATc1 mRNA expression status (**F**) or co-alteration of SMAD4 and NFATc1 (**G**). *P ≤ 0.05 from Wilcoxon rank-sum test. The correlation between STAT3 and NFATc1 expression was determined in SMAD4-low (**H**) and SMAD4-high (**I**) TCGA-PDAC patient samples. SMAD4- and NFATc1-high/low groups were defined based on the top and bottom one-third of mRNA expression levels across the cohort with tumor sample purity >25%. Pearson correlation analysis was performed. (**J** and **K**) Upregulation of STAT3 protein levels in SMAD4-KD MIA-PaCa2 (**J**) and Panc-1 (**K**) cells. (**L** and **M**) NFATc1 inhibition attenuates SMAD4 KD-induced STAT3 upregulation. SMAD4-KD MIA-PaCa2 (**L**) and Panc-1 (**M**) cells were treated with Iono+PMA for 4 h or CsA for 1 h. (**N** and **O**) NFATc1 KD reduces STAT3 protein levels in SMAD4-KD MIA-PaCa2 (**N**) and Panc-1 (**O**) cells. Data are presented as representative immunoblots from three independent experiments.

Our findings that the SMAD4–NFATc1 interaction is mediated by SMAD4’s MH1 domain and is preserved in SMAD3 binding-deficient SMAD4 mutants led us to hypothesize that this PPI operates independently of TGF-**β** signaling. To test this hypothesis, we examined the SMAD4–NFATc1 PPI under conditions of pharmacological activation and inhibition of TGF-**β** signaling using human recombinant TGF-**β**1 protein and SB431542, a TGF-**β** receptor kinase inhibitor, respectively. As expected, the SMAD4–SMAD3 PPI was significantly reduced upon inhibition of TGF-**β** signaling, confirming the canonical TGF-**β** dependency, while the SMAD4–NFATc1 PPI remained unchanged regardless of TGF-**β** signaling activation or inhibition, supporting that this interaction is independent of TGF-**β** signaling (Figure 5A).

The observed TGF-**β**-independent nature of the SMAD4–NFATc1 PPI prompted us to investigate its functional significance in regulating NFATc1 activity. Under basal conditions, NFATc1 is phosphorylated and retained in the cytoplasm (Macian, 2005). Upon activation, NFATc1 is dephosphorylated and translocates into the nucleus to drive gene transcription (Macian, 2005). To determine whether the SMAD4–NFATc1 interaction is dependent on NFATc1’s phosphorylation state, we treated cells with ionomycin and phorbol myristate acetate (Iono+PMA) to activate NFATc1 or cyclosporin A (CsA) to inhibit NFATc1 (Loh et al., 1996). CsA treatment remarkably increased NFATc1 phosphorylation and enhanced interaction between VF-NFATc1 and SMAD4, compared to Iono+PMA treatment (Figure 5B). These results indicate that SMAD4 preferentially binds to the phosphorylated NFATc1.

Next, we performed an NFAT-luciferase reporter assay to assess NFATc1 transcriptional activity. As a positive control, CsA treatment attenuated NFAT reporter activity by 25%, while overexpression of VF-SMAD4 reduced NFAT reporter activity by 45% (Figure 5C). Along with the findings that the SMAD4–NFATc1 interaction predominantly localizes to the cytoplasm (Figure 3D) and SMAD4 binds to the inactive phosphorylated (cytoplasmic) NFATc1 (Figure 5B), these results suggest that SMAD4 inhibits NFATc1 transcriptional activity, likely by sequestering the phosphorylated NFATc1 in the cytoplasm and preventing its nuclear translocation.

### Identification of the SMAD4–NFATc1–STAT3 regulatory axis in PDAC cells

Given that NFATc1 emerged as a top-ranked master regulator with upregulated transcriptional activity in SMAD4-KD PDAC cells (Figure 2E), we hypothesized that the loss of the inhibitory SMAD4–NFATc1 OncoPPI complex might release NFATc1 to activate oncogenic target genes in SMAD4-deficient PDAC cells. To identify potential NFATc1 target genes, we revisited our differential transcriptome (Figure 2A) and proteome (Figure 2D) datasets. These unbiased multi-omics analyses revealed that STAT3 was one of the most upregulated genes (Figure 5D) and proteins (Figure 5E) in SMAD4-KD MIA-PaCa2 cells compared to their WT counterparts, suggesting that STAT3 may be a direct target of NFATc1 in SMAD4-deficient PDAC cells.

Then, we analyzed the correlation between NFATc1 and STAT3 expression in the TCGA-PDAC cohort. STAT3 mRNA levels were significantly elevated in NFATc1-high PDAC samples, indicating a positive correlation between NFATc1 and STAT3 expression (Figure 5F). Notably, this correlation was only evident in samples with low SMAD4 expression but absent in samples with high SMAD4 expression (Figure 5G). In SMAD4-low PDAC samples, NFATc1 and STAT3 mRNA levels displayed a statistically significant positive correlation (*R* = 0.37, *P* = 0.043) (Figure 5H). By contrast, in SMAD4-high samples, the correlation between NFATc1 and STAT3 was not significant (*R* = 0.17, *P* = 0.36) (Figure 5I). These results highlight a clinically relevant SMAD4–NFATc1–STAT3 regulatory axis in SMAD4-deficient PDAC cells, implicating STAT3 as a potential NFATc1-regulated gene.

To validate this regulatory axis, we conducted pharmacological and genetic perturbation studies in PDAC cell line models. We confirmed that STAT3 protein levels were significantly upregulated in isogenic SMAD4-KD MIA-PaCa2 (Figure 5J) and Panc-1 (Figure 5K) cells. In SMAD4-KD MIA-PaCa2 cells, pharmacological inhibition of NFATc1 using CsA significantly reduced STAT3 protein expression, while activation of NFATc1 with Iono+PMA restored STAT3 protein levels (Figure 5L). In addition, NFATc1 KD in SMAD4-KD MIA-PaCa2 cells resulted in a marked reduction of STAT3 protein level (Figure 5N). These results were further corroborated in SMAD4-KD Panc-1 cells (Figure 5M and O). However, in SMAD4-WT cells, we did not observe significant change of STAT3 mRNA level upon NFATc1 KD (Supplementary Figure S1D). Furthermore, we performed ChIP–qPCR assay and found a significantly increased enrichment of NFATc1 occupancy in the STAT3 promoter region in the SMAD4-KD MIA-PaCa2 cells compared to the WT counterparts (Supplementary Figure S1E). Collectively, these data suggest NFATc1 as a key upstream regulator of STAT3 expression in SMAD4-deficient PDAC cells, supporting a rewired SMAD4–NFATc1–STAT3 regulatory axis with potential therapeutic implications.

### Therapeutic vulnerability of targeting STAT3 in SMAD4-KD PDAC cells

**Figure 6:**
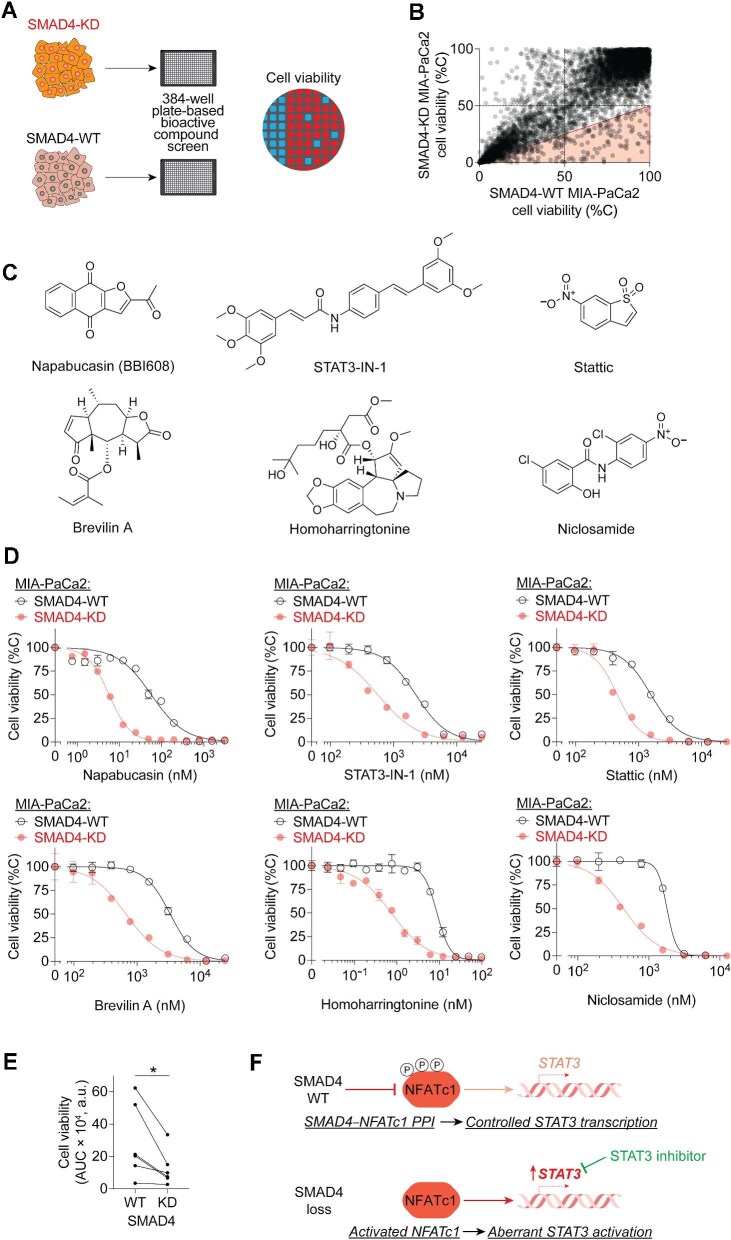
Identification of STAT3 as a therapeutic vulnerability in SMAD4-KD PDAC cells. (**A**) Differential drug sensitivity screening using isogenic SMAD4-WT and SMAD4-KD MIA-PaCa2 cells. (**B**) Drug sensitivity for each compound in SMAD4-WT and SMAD4-KD MIA-PaCa2 cells. Data are presented as single data points from the primary screening. (**C**) Chemical structures of six identified STAT3 inhibitors. (**D** and **E**) Dose–response curves (**D**) and the area-under-the-curve (AUC) quantification of cell viability (**E**) showing STAT3 inhibitor-induced growth inhibition of SMAD4-WT and SMAD4-KD MIA-PaCa2 cells. Data in dose–response curves are presented as mean ± SD from three independent experiments. In the paired dot plot, each line represents a STAT3 inhibitor. *P ≤ 0.05 by paired *t*-test. (**F**) Schematic diagram of the rewired SMAD4–NFATc1–STAT3 axis and collateral vulnerability in SMAD4-deficient PDAC cells.

To investigate potential therapeutic vulnerabilities in SMAD4-deficient PDAC cells, we conducted an unbiased chemogenomic screen using isogenic SMAD4-KD MIA-PaCa2 cells. The screening was performed in a high-throughput 384-well plate format against the Emory Enriched Bioactive Library (EEBL) (Mo et al., 2019), which contains 12807 bioactive small molecules with annotated biological targets or pathways (Figure 6A). This cell viability-based screen identified 142 compounds that demonstrated >2-fold higher anti-tumor activity in SMAD4-KD cells compared to their WT counterparts (Figure 6B).

Among the hits selectively targeting SMAD4-KD cells, six structurally distinct small molecules were identified as inhibitors of STAT3 or the JAK–STAT pathway. These compounds included Napabucasin (BBI608), STAT3-IN-1, Stattic, Brevilin A, Homoharringtonine, and Niclosamide (Figure 6C). To validate their selectivity, we performed dose–response viability assays. For example, Napabucasin exhibited dose-dependent inhibition of SMAD4-KD MIA-PaCa2 cells, with significantly greater potency (IC_50_ = 5.71 ± 1.05 nM) compared to WT cells (IC_50_ = 57.1 ± 10.6 nM) (Figure 6D). Similarly, the other five inhibitors all demonstrated selective anti-proliferative activity against SMAD4-KD cells over WT cells (Figure 6D and E). These findings suggest that SMAD4-deficient PDAC cells exhibit a dependency on STAT3, potentially driven by the rewired SMAD4–NFATc1–STAT3 axis, presenting a therapeutically targetable vulnerability.

In summary, our findings reveal an SMAD4–NFATc1 PPI that operates independently of canonical TGF-**β** signaling: SMAD4 suppresses NFATc1 transcriptional activity in SMAD4-WT cells through binding to and sequestering the phosphorylated and inactive form of NFATc1 in a TGF-**β**-independent manner, while the loss of SMAD4–NFATc1 PPI in SMAD4-deficient PDAC cells leads to the activation of NFATc1-mediated STAT3 expression, presenting a potential therapeutic vulnerability (Figure 6F). These results pave the way for future studies to examine the clinical relevance of targeting the SMAD4–NFATc1–STAT3 axis and to develop innovative strategies for personalized cancer therapy.

## Discussion

We report a distinct SMAD4–NFATc1 OncoPPI that operates independently of the canonical SMAD4–SMAD3 complex within the TGF-**β** signaling pathway (Zhao et al., 2018). While SMAD4 is regarded as a central mediator of TGF-**β** signaling (Nakao et al., 1997), our findings highlight a non-canonical role for SMAD4 in regulating NFATc1. Specifically, the SMAD4–NFATc1 interaction sequesters NFATc1 in the cytoplasm, limiting its nuclear translocation and transcriptional activity. This mechanism is distinct from canonical SMAD4-mediated transcriptional regulation (Nakao et al., 1997) and underscores a novel TGF-**β**-independent function of SMAD4. The validation of the SMAD4–NFATc1 PPI in Jurkat T cells suggests its potential significance in regulating T cell functions. In SMAD4-deficient cancer cells, disruption of this interaction liberates NFATc1, enabling activation of oncogenic pathways such as STAT3 signaling. These findings expand the understanding of SMAD4’s role in maintaining cellular homeostasis and suggest broader implications for its TGF-**β**-independent functions in disease biology.

PDAC remains one of the most lethal malignancies, characterized by an exceptionally poor prognosis and limited therapeutic options. The genomic landscape of PDAC is dominated by mutations in key driver genes, including *KRAS, TP53, CDKN2A*, and *SMAD4*, which collectively drive tumorigenic pathways that are notoriously difficult to target (Hayashi et al., 2021). Among these, *SMAD4* mutations, present in >50% of cases, represent a critical transition in tumor progression and are associated with increased invasiveness and poor outcomes (Fei et al., 2021; Principe et al., 2022), yet their detailed molecular mechanisms remain poorly understood. This study identifies a regulatory axis involving SMAD4, NFATc1, and STAT3, which not only broadens the functional repertoire of SMAD4 but also reveals a novel therapeutic vulnerability in PDAC (Hahn et al., 2021).

The identification of STAT3 as a downstream effector of the SMAD4–NFATc1 axis provides mechanistic insights into the aggressive phenotype observed in SMAD4-deficient PDAC. STAT3, a well-established oncogenic transcription factor, is frequently activated in PDAC and plays critical roles in cell proliferation, invasion, immune evasion, and cancer stemness (Zhao et al., 2008; Hamel et al., 2024). Our study demonstrates that SMAD4 loss enables NFATc1 to drive STAT3 expression, creating a dependency that fuels tumor progression. Importantly, SMAD4-deficient PDAC cells exhibit heightened sensitivity to STAT3 inhibition, highlighting this axis as a compelling therapeutic target. Small-molecule STAT3 inhibitors and antisense oligonucleotides, some of which are currently undergoing clinical evaluation, showed promising efficacy in PDAC patients (Hamel et al., 2024). Our study implicates that the combination of SMAD4 mutation status and elevated NFATc1 expression could serve as a surrogate biomarker to identify patients most likely to benefit from STAT3-targeted therapies. These findings emphasize the translational potential of STAT3 inhibitors and the critical role of molecular biomarkers in precision medicine strategies for PDAC.

The SMAD4–NFATc1–STAT3 axis exemplifies the complex oncogenic rewiring driven by SMAD4 loss in PDAC. SMAD4 deficiency has been implicated in various signaling alterations, each contributing to unique vulnerabilities and resistance mechanisms. For instance, SMAD4 loss enhances EGFR and ERBB2 expression in cooperation with oncogenic KRAS, promoting invasiveness and progression (Zhao et al., 2010; Liu et al., 2015). SMAD4 loss also activates metastatic drivers like FOSL1, facilitating colonization in distant microenvironments (Dai et al., 2021). Additionally, SMAD4 loss disrupts its interaction with PARP1, increasing DNA damage repair efficiency and rendering PDAC resistant to radiotherapy. Synthetic lethality approaches using PARP inhibitors such as olaparib have shown promise in overcoming this resistance (Wang et al., 2024). Our findings extend this framework by uncovering the SMAD4–NFATc1–STAT3 regulatory axis as a novel signaling pathway linking SMAD4 loss to STAT3 activation, further illustrating the versatility of SMAD4-deficient PDAC in leveraging diverse pathways for survival and metastasis. These insights identify actionable vulnerabilities, such as STAT3 dependency, that can be harnessed for precision therapeutic strategies.

NFAT transcription factors, particularly NFATc1, play pivotal roles in driving PDAC progression through diverse oncogenic mechanisms (Buchholz et al., 2006; Baumgart et al., 2014; Hasselluhn et al., 2019). Previous studies demonstrated that NFATc1 interacts with SMAD3 and cJUN in SMAD4-deficient tumors to form transcriptional complexes that enhance chemoresistance and aggressiveness (Hasselluhn et al., 2024). Our findings independently complement this by revealing a previously uncharacterized SMAD3-independent interaction between SMAD4 and NFATc1, establishing a distinct molecular link between SMAD4 and NFATc1’s oncogenic functions. Furthermore, while NFATc1 has been shown to antagonize TGF-**β** signaling (Hasselluhn et al., 2019), our results indicate that the SMAD4–NFATc1 interaction provides a complementary layer of regulation. This mechanism may reshape transcriptional landscapes in SMAD4-deficient PDAC (Yao et al., 2024).

Additionally, NFATc1 directly interacts with STAT3 to amplify inflammatory signaling and promote chemoresistance (Baumgart et al., 2014). Our study connects this observation to transcriptional regulation, demonstrating that NFATc1 directly upregulates STAT3 expression in SMAD4-deficient tumors, thereby reinforcing the dependency on STAT3 activity. This novel link highlights NFATc1’s versatility and establishes the SMAD4–NFATc1–STAT3 axis as a critical driver of PDAC oncogenesis. While outside the scope of this study, other NFAT family members, such as NFAT5, have been implicated in KRAS inhibitor resistance through roles in EMT and tumor plasticity (Deng et al., 2024). Exploring the intersection of NFAT-, SMAD4-, and KRAS-mediated pathways represents an exciting avenue for future research, particularly given the overlap between transcriptional rewiring and therapeutic resistance in PDAC.

In conclusion, this study identifies the SMAD4–NFATc1–STAT3 axis as a critical mediator of oncogenesis in SMAD4-deficient PDAC, offering novel insights into how the loss of a tumor suppressor rewires transcriptional networks to drive tumor progression (Fu et al., 2025). By establishing STAT3 as a key dependency in this context, our findings underscore a promising therapeutic target and enhance the broader understanding of PDAC biology. While this study provides compelling *in vitro* evidence, further validation using patient-derived xenografts or genetically engineered mouse models will be essential to examine these findings in physiologically relevant settings. Such efforts will improve the translational impact of this work and support the development of targeted therapeutic strategies for SMAD4-deficient PDAC.

## Materials and methods

### Cell culture

All cell lines were incubated at 37°C in humidified conditions with 5% CO_2_. Human embryonic kidney 293T cells (HEK293T; ATCC, CRL-3216) were maintained in Dulbecco's Modified Eagle's Medium (DMEM; Corning, #10-013-CV). Human pancreatic adenocarcinoma cell lines, including MIA-PaCa2 (ATCC, CRL-1420), Capan-2 (ATCC, HTB-80), and Panc-1 (ATCC, CRL-1469), were maintained in DMEM. The acute T-cell leukemia cell line Jurkat (ATCC, TIB-152) was maintained in RPMI-1640 Medium (Corning, #10-040-CV). Cell culture medium was supplemented with 10% fetal bovine serum (FBS; ATLANTA biologicals, #S11550) and 100 units/ml of penicillin/streptomycin (Cell Gro, Cat# 30-002-CI).

### Plasmids

Plasmids for mammalian expression of GST- (Li et al., 2017), NLuc-, VF- (Li et al., 2017), and N- or C-Venus-tagged (Mo et al., 2022) proteins were generated using the Gateway cloning system (Invitrogen) according to the manufacturer's protocol. The SMAD4, NFATc1, and other library genes in pDONR were purchased from the Human ORFeome V8.1 set. The 556 VF-tagged OncoPPI gene library were cloned as reported previously (Mo et al., 2022). The SMAD4 and NFATc1 domain truncations in pDONR223 were generated using PCR and the Gateway cloning system. The SMAD4 and NFATc1 mutations were introduced using the QuikChange Lightning Site-Directed Mutagenesis Kit (Agilent Technologies, Cat# 210518). Plasmids for shRNA KD were purchased from MISSON shRNA library (Sigma-Aldrich). All the plasmids were confirmed by sequencing.

### SMAD4 OncoPPI mapping

The SMAD4 OncoPPI mapping was performed using our previously established BRET-based (Mo et al., 2022) and TR-FRET-based (Li et al., 2017; Xiong et al., 2018; Ouyang et al., 2023; Lindsay et al., 2025) PPI screening technology as detailed in Supplementary material.

### GST-pulldown assay

Cell lysate-based affinity-pulldown assay was performed as previously described (Mo and Fu, 2016; Mo et al., 2016, 2017; Li et al., 2017; Boettcher et al., 2018; Grzeskowiak et al., 2018; Ivanov et al., 2018; Rusnak et al., 2018; Tang et al., 2021). See details in Supplementary material.

### Co-IP with endogenous proteins

The endogenous co-IP assay was performed as described previously (Mo et al., 2017; Rusnak et al., 2018; Tang et al., 2021). See details in Supplementary material.

### BiFC

The BiFC was performed as described previously (Mo et al., 2022). See details in Supplementary material.

### NFAT luciferase reporter assay

HEK293T cells were grown in 6-well plates and transfected using FuGene with corresponding VF-SMAD4 or VF-empty control plasmids, along with pGL3-NFAT luciferase (Addgene Plasmid #17870; Clipstone and Crabtree, 1992). Renilla luciferase was included as an internal control. After transfection, cells were incubated for 48 h in DMEM supplemented with 10% FBS. Cells were harvested mechanically, centrifuged at 1600 rpm for 2 min, and then resuspended in 300 ml of DMEM. The cells were transferred to the 384-well plate, and the reporter assay was performed using the Dual-Glo Luciferase Kit (Promega, E2920) following the manufacturer's instructions. Firefly luciferase expression was normalized to the internal control Renilla expression.

### Transcriptome (RNA-seq) analysis

The SMAD4 KD-induced transcriptomic changes were analyzed by mRNA sequencing service at Novogene Corporation Inc. Briefly, the total RNA from the isogenic SMAD4-WT and SMAD4-KD MIA-PaCa2 cells was isolated using E.Z.N.A.^®^ Total RNA Kit I. RNA-seq reads were aligned to the human reference genome (GRCh38). Significantly up- or downregulated DEGs were identified using |log_2_(fold change)| ≥ 1 and adjusted *P*-value ≤0.05. Pathway enrichment analysis was performed using DESeq R packages (Zhou et al., 2019).

### Bioinformatics analysis

GSEA was performed as described previously (Tang et al., 2021). Briefly, the GSEABase package in R Studio was used to score the indicating gene sets. The Hallmark gene sets available from the MSigDB (Liberzon et al., 2015) were used as the reference gene sets. The rank of genes in the indicating pathways was used in accordance with the DEGs identified between isogenic cell lines. The normalized enrichment score (NES) was calculated to reflect the degree to which a set of genes is overrepresented at the extremes (top or bottom) among the entire ranked list. |NES| > 1 with the *P*-value and false discovery rate (FDR) <0.05 was considered as significantly enriched. The DEGs were subjected to the transcriptional master regulator analysis using VIPER algorithm (Alvarez et al., 2016). All relevant data for TCGA-PDAC analysis, including transcriptomics, clinical, and tumor purity data, were obtained from the pdacR package (Torre-Healy et al., 2023), which contains a total of 181 samples with 150 whitelisted by histologic identification of neoplastic cellularity and cancer type. The 56 samples with absolute purity <25% were filtered out from downstream analysis. The two-sample *t*-test or Spearman correlation test was used for statistical correlation analysis.

### HTS cell viability assay

Cells were seeded at 3000 cells/well in 50 μl media in the 384-well culture plate (Corning, Cat#3764) using a Multidrop^TM^ Combi Reagent Dispenser (Thermo Scientific) with the first column as a medium-only control (Blk). The next day, test compounds (0.1 μl) were dispensed into wells in each plate using a Biomek NX^P^ Lab Automation Workstation (Beckman Coulter) from a compound stock plate to give the indicated final concentrations. After 3 days of incubation, 10 μl CellTiter-Blue (Promega, G8081) was added to each well using Multidrop^TM^ Combi Reagent Dispenser (Thermo Scientific). The plate was incubated for 1–4 h at 37°C. The fluorescence intensity (FI) of each well was read using a BMG PHERASTAR plate reader (Ex 545 nm and Em 615 nm). Percentage of Control (%C) was calculated using the equation: (FI_compound_ − FI_Avg. Blk_)/(FI_Avg. Neg._ − FI_Avg. Blk_) × 100.

### Statistics and reproducibility

The BRET OncoPPI screening data were quantified and analyzed using the CARINA algorithm (Mo et al., 2022). Biochemical and biological assays were performed and repeated three times. All analyses were performed using GraphPad Prism version 7.0 (GraphPad Software) or Microsoft Excel. The dose-dependent small molecule-induced cancer cell growth inhibition curve was established using GraphPad Prism based on the sigmoidal dose–response (variable slope) equation. Statistical significance was assessed using Student's *t*-test or Wilcoxin test, as indicated. Statistical tests with exact *P*-values derived, the number of independent biological replicates, and, where applicable, the number of analyzed cells are provided in the figures and figure legends. *P* ≤ 0.05 was considered statistically significant. No statistical method was used to predetermine the sample size. No data were excluded from the analyses. The experiments were not randomized. The investigators were not blinded to allocation during experiments and outcome assessment.

## Supplementary Material

mjaf028_Supplemental_File

## Data Availability

The mass spectrometry proteomics data files are deposited to ProteomeXchange Consortium via the PRIDE partner repository (Vizcaino et al., 2014) and are available with the data identifier PXD067749.

## Notes

**Author contributions:** X.M. conceptualized the project. W.O. and Q.N. performed *in vitro* molecular and cellular biology studies. X.M., Q.N., and Y.D. performed the OncoPPI and chemical screen. J.H., E.D., M.H., and R.M. performed bioinformatics analysis. C.M.B., and D.E.G. performed proteomics analysis. W.O. and X.M. wrote the initial manuscript. X.M. organized resources, fundings, and project coordination and supervision. All authors reviewed and edited the manuscript.

## References

[bib1] Alvarez M.J., Shen Y., Giorgi F.M. et al. (2016). Functional characterization of somatic mutations in cancer using network-based inference of protein activity. Nat. Genet. 48, 838–847. 27322546 10.1038/ng.3593 PMC5040167

[bib2] Baumgart S., Chen N.M., Siveke J.T. et al. (2014). Inflammation-induced NFATc1–STAT3 transcription complex promotes pancreatic cancer initiation by KrasG12D. Cancer Discov. 4, 688–701. 24694735 10.1158/2159-8290.CD-13-0593 PMC4069603

[bib3] Boettcher M., Tian R., Blau J.A. et al. (2018). Dual gene activation and knockout screen reveals directional dependencies in genetic networks. Nat. Biotechnol. 36, 170–178. 29334369 10.1038/nbt.4062 PMC6072461

[bib4] Buchholz M., Schatz A., Wagner M. et al. (2006). Overexpression of c-myc in pancreatic cancer caused by ectopic activation of NFATc1 and the Ca^2+^/calcineurin signaling pathway. EMBO J. 25, 3714–3724. 16874304 10.1038/sj.emboj.7601246 PMC1538549

[bib5] Chandiran K., Cauley L.S. (2023). The diverse effects of transforming growth factor-β and SMAD signaling pathways during the CTL response. Front. Immunol. 14, 1199671. 37426662 10.3389/fimmu.2023.1199671 PMC10327426

[bib6] Chen L., Glover J.N., Hogan P.G. et al. (1998). Structure of the DNA-binding domains from NFAT, Fos and Jun bound specifically to DNA. Nature 392, 42–48. 9510247 10.1038/32100

[bib7] Clipstone N.A., Crabtree G.R. (1992). Identification of calcineurin as a key signalling enzyme in T-lymphocyte activation. Nature 357, 695–697. 1377362 10.1038/357695a0

[bib8] Cox A.D., Der C.J. (2024). ‘Undruggable KRAS’: druggable after all. Genes Dev. 39, 132–162. 10.1101/gad.352081.124 PMC11789494 39638567

[bib9] Dai C., Rennhack J.P., Arnoff T.E. et al. (2021). SMAD4 represses FOSL1 expression and pancreatic cancer metastatic colonization. Cell Rep. 36, 109443. 34320363 10.1016/j.celrep.2021.109443 PMC8350598

[bib10] de Caestecker M.P., Hemmati P., Larisch-Bloch S. et al. (1997). Characterization of functional domains within Smad4/DPC4. J. Biol. Chem. 272, 13690–13696. 9153220 10.1074/jbc.272.21.13690

[bib11] Deng D., Begum H., Liu T. et al. (2024). NFAT5 governs cellular plasticity-driven resistance to KRAS-targeted therapy in pancreatic cancer. J. Exp. Med. 221, e20240766. 39432061 10.1084/jem.20240766 PMC11497412

[bib12] Dong X.M., Yin R.H., Yang Y. et al. (2014). GATA-2 inhibits transforming growth factor-β signaling pathway through interaction with Smad4. Cell. Signal. 26, 1089–1097. 24509415 10.1016/j.cellsig.2014.01.028

[bib13] Fei N., Wen S., Ramanathan R. et al. (2021). SMAD4 loss is associated with response to neoadjuvant chemotherapy plus hydroxychloroquine in patients with pancreatic adenocarcinoma. Clin. Transl. Sci. 14, 1822–1829. 34002944 10.1111/cts.13029 PMC8504806

[bib14] Fu H., Mo X., Ivanov A.A. (2025). Decoding the functional impact of the cancer genome through protein–protein interactions. Nat. Rev. Cancer 25, 189–208. 39810024 10.1038/s41568-024-00784-6 PMC12291072

[bib15] Gowripalan A., Abbott C.R., McKenzie C. et al. (2020). Cell-to-cell spread of vaccinia virus is promoted by TGF-**β**-independent Smad4 signalling. Cell. Microbiol. 22, e13206. 32237038 10.1111/cmi.13206

[bib16] Grzeskowiak C.L., Kundu S.T., Mo X. et al. (2018). In vivo screening identifies GATAD2B as a metastasis driver in KRAS-driven lung cancer. Nat. Commun. 9, 2732. 30013058 10.1038/s41467-018-04572-3 PMC6048166

[bib17] Gutierrez M.L., Munoz-Bellvis L., Orfao A. (2021). Genomic heterogeneity of pancreatic ductal adenocarcinoma and its clinical impact. Cancers 13, 4451. 34503261 10.3390/cancers13174451 PMC8430663

[bib18] Hahn W.C., Bader J.S., Braun T.P. et al. (2021). An expanded universe of cancer targets. Cell 184, 1142–1155. 33667368 10.1016/j.cell.2021.02.020 PMC8066437

[bib19] Hamel Z., Sanchez S., Standing D. et al. (2024). Role of STAT3 in pancreatic cancer. Explor. Target Antitumor Ther. 5, 20–33. 38464736 10.37349/etat.2024.00202 PMC10918236

[bib20] Hasselluhn M.C., Schlosser D., Versemann L. et al. (2024). An NFATc1/SMAD3/cJUN complex restricted to SMAD4-deficient pancreatic cancer guides rational therapies. Gastroenterology 166, 298–312.e14.37913894 10.1053/j.gastro.2023.10.026

[bib21] Hasselluhn M.C., Schmidt G.E., Ellenrieder V. et al. (2019). Aberrant NFATc1 signaling counteracts TGFβ-mediated growth arrest and apoptosis induction in pancreatic cancer progression. Cell Death. Dis. 10, 446. 31171768 10.1038/s41419-019-1682-2 PMC6554303

[bib22] Hayashi A., Hong J., Iacobuzio-Donahue C.A. (2021). The pancreatic cancer genome revisited. Nat. Rev. Gastroenterol. Hepatol. 18, 469–481. 34089011 10.1038/s41575-021-00463-z

[bib23] Hosein A.N., Brekken R.A., Maitra A. (2020). Pancreatic cancer stroma: an update on therapeutic targeting strategies. Nat. Rev. Gastroenterol. Hepatol. 17, 487–505. 32393771 10.1038/s41575-020-0300-1 PMC8284850

[bib24] Igalouzene R., Hernandez-Vargas H., Benech N. et al. (2022). SMAD4 TGF-**β**-independent function preconditions naive CD8^+^ T cells to prevent severe chronic intestinal inflammation. J. Clin. Invest. 132, e151020. 35426367 10.1172/JCI151020 PMC9012287

[bib25] Ivanov A.A., Revennaugh B., Rusnak L. et al. (2018). The OncoPPi Portal: an integrative resource to explore and prioritize protein–protein interactions for cancer target discovery. Bioinformatics 34, 1183–1191. 29186335 10.1093/bioinformatics/btx743 PMC6030952

[bib26] Jain J., McCaffrey P.G., Miner Z. et al. (1993). The T-cell transcription factor NFATp is a substrate for calcineurin and interacts with Fos and Jun. Nature 365, 352–355. 8397339 10.1038/365352a0

[bib27] Kleeff J., Korc M., Apte M. et al. (2016). Pancreatic cancer. Nat. Rev. Dis. Primers 2, 16022. 27158978 10.1038/nrdp.2016.22

[bib28] Li Z., Ivanov A.A., Su R. et al. (2017). The OncoPPi network of cancer-focused protein–protein interactions to inform biological insights and therapeutic strategies. Nat. Commun. 8, 14356. 28205554 10.1038/ncomms14356 PMC5316855

[bib29] Liberzon A., Birger C., Thorvaldsdottir H. et al. (2015). The Molecular Signatures Database (MSigDB) hallmark gene set collection. Cell Syst. 1, 417–425. 26771021 10.1016/j.cels.2015.12.004 PMC4707969

[bib30] Lindsay M.E., Scimone E.R., Lawton J. et al. (2025). Gain-of-function variants in SMAD4 compromise respiratory epithelial function. J. Allergy Clin. Immunol. 155, 107–119.e2. 39243984 10.1016/j.jaci.2024.08.024 PMC11700783

[bib31] Liu J., Cho S.N., Akkanti B. et al. (2015). ErbB2 pathway activation upon Smad4 loss promotes lung tumor growth and metastasis. Cell Rep. 10, 1599–1613. 25753424 10.1016/j.celrep.2015.02.014 PMC7405934

[bib32] Loh C., Carew J.A., Kim J. et al. (1996). T-cell receptor stimulation elicits an early phase of activation and a later phase of deactivation of the transcription factor NFAT1. Mol. Cell. Biol. 16, 3945–3954. 8668212 10.1128/mcb.16.7.3945 PMC231391

[bib33] Macian F. (2005). NFAT proteins: key regulators of T-cell development and function. Nat. Rev. Immunol. 5, 472–484. 15928679 10.1038/nri1632

[bib34] Macian F., Garcia-Rodriguez C., Rao A. (2000). Gene expression elicited by NFAT in the presence or absence of cooperative recruitment of Fos and Jun. EMBO J. 19, 4783–4795. 10970869 10.1093/emboj/19.17.4783 PMC302068

[bib35] Mo X., Fu H. (2016). BRET: NanoLuc-based bioluminescence resonance energy transfer platform to monitor protein–protein interactions in live cells. In: Janzen W.P. (ed). High Throughput Screening: Methods and Protocols. New York, NY: Springer, 263–271. 10.1007/978-1-4939-3673-1_17 27317001

[bib36] Mo X., Luo Y., Ivanov A.A. et al. (2016). Enabling systematic interrogation of protein–protein interactions in live cells with a versatile ultra-high-throughput biosensor platform. J. Mol. Cell Biol. 8, 271–281. 26578655 10.1093/jmcb/mjv064 PMC4937889

[bib37] Mo X., Niu Q., Ivanov A.A. et al. (2022). Systematic discovery of mutation-directed neo-protein–protein interactions in cancer. Cell 185, 1974–1985.e12. 35512704 10.1016/j.cell.2022.04.014 PMC9597701

[bib38] Mo X., Qi Q., Ivanov A.A. et al. (2017). AKT1, LKB1, and YAP1 revealed as MYC interactors with NanoLuc-based protein-fragment complementation assay. Mol. Pharmacol. 91, 339–347. 28087810 10.1124/mol.116.107623 PMC5363710

[bib39] Mo X., Tang C., Niu Q. et al. (2019). HTiP: high-throughput immunomodulator phenotypic screening platform to reveal IAP antagonists as anti-cancer immune enhancers. Cell Chem. Biol. 26, 331–339.e3. 30639259 10.1016/j.chembiol.2018.11.011 PMC6501824

[bib40] Nakao A., Imamura T., Souchelnytskyi S. et al. (1997). TGF-**β** receptor-mediated signalling through Smad2, Smad3 and Smad4. EMBO J. 16, 5353–5362. 9311995 10.1093/emboj/16.17.5353 PMC1170167

[bib41] Ouyang W., Li Q., Niu Q. et al. (2023). A multiplexed time-resolved fluorescence resonance energy transfer ultrahigh-throughput screening assay for targeting the SMAD4–SMAD3–DNA complex. J. Mol. Cell Biol. 15, mjad068. 10.1093/jmcb/mjad068 PMC11063955 37968137

[bib42] Park W., Chawla A., O'Reilly E.M. (2021). Pancreatic cancer: a review. JAMA 326, 851–862. 34547082 10.1001/jama.2021.13027 PMC9363152

[bib43] Principe D.R., Underwood P.W., Kumar S. et al. (2022). Loss of SMAD4 is associated with poor tumor immunogenicity and reduced PD-L1 expression in pancreatic cancer. Front. Oncol. 12, 806963. 35155243 10.3389/fonc.2022.806963 PMC8832494

[bib44] Rusnak L., Tang C., Qi Q. et al. (2018). Large tumor suppressor 2, LATS2, activates JNK in a kinase-independent mechanism through ASK1. J. Mol. Cell Biol. 10, 549–558. 30496488 10.1093/jmcb/mjy061 PMC7191879

[bib45] Seoane J., Le H.V., Shen L. et al. (2004). Integration of Smad and forkhead pathways in the control of neuroepithelial and glioblastoma cell proliferation. Cell 117, 211–223. 15084259 10.1016/s0092-8674(04)00298-3

[bib46] Shugang X., Hongfa Y., Jianpeng L. et al. (2016). Prognostic value of SMAD4 in pancreatic cancer: a meta-analysis. Transl. Oncol. 9, 1–7. 26947875 10.1016/j.tranon.2015.11.007 PMC4800056

[bib47] Song J., Wu J., Ding J. et al. (2023). The effect of SMAD4 on the prognosis and immune response in hypopharyngeal carcinoma. Front. Med. 10, 1139203. 10.3389/fmed.2023.1139203 PMC10076535 37035326

[bib48] Tang C., Mo X., Niu Q. et al. (2021). Hypomorph mutation-directed small-molecule protein–protein interaction inducers to restore mutant SMAD4-suppressed TGF-**β** signaling. Cell Chem. Biol. 28, 636–647.e5. 33326750 10.1016/j.chembiol.2020.11.010 PMC10053325

[bib49] Timmer F.E.F., Geboers B., Nieuwenhuizen S. et al. (2021). Pancreatic cancer and immunotherapy: a clinical overview. Cancers 13, 4138. 34439292 10.3390/cancers13164138 PMC8393975

[bib50] Torre-Healy L.A., Kawalerski R.R., Oh K. et al. (2023). Open-source curation of a pancreatic ductal adenocarcinoma gene expression analysis platform (pdacR) supports a two-subtype model. Commun. Biol. 6, 163. 36765128 10.1038/s42003-023-04461-6 PMC9918476

[bib51] Vizcaino J.A., Deutsch E.W., Wang R. et al. (2014). ProteomeXchange provides globally coordinated proteomics data submission and dissemination. Nat. Biotechnol. 32, 223–226. 24727771 10.1038/nbt.2839 PMC3986813

[bib52] Wang F., Xia X., Yang C. et al. (2018a). SMAD4 gene mutation renders pancreatic cancer resistance to radiotherapy through promotion of autophagy. Clin. Cancer Res. 24, 3176–3185. 29602802 10.1158/1078-0432.CCR-17-3435 PMC6345154

[bib53] Wang Y., Chu J., Yi P. et al. (2018b). SMAD4 promotes TGF-**β**-independent NK cell homeostasis and maturation and antitumor immunity. J. Clin. Invest. 128, 5123–5136. 30183689 10.1172/JCI121227 PMC6205382

[bib54] Wang Y., Yu T., Zhao Z. et al. (2024). SMAD4 limits PARP1 dependent DNA repair to render pancreatic cancer cells sensitive to radiotherapy. Cell Death. Dis. 15, 818. 39528473 10.1038/s41419-024-07210-7 PMC11555233

[bib55] Xiong J., Pecchi V.G., Qui M. et al. (2018). Development of a time-resolved fluorescence resonance energy transfer ultrahigh-throughput screening assay for targeting the NSD3 and MYC interaction. Assay Drug Dev. Technol. 16, 96–106. 29634317 10.1089/adt.2017.835 PMC5865254

[bib56] Yao H., Luo L., Li R. et al. (2024). New insight into the role of SMAD4 mutation/deficiency in the prognosis and therapeutic resistance of pancreatic ductal adenocarcinomas. Bioch. Biophys. Acta Rev. Cancer 1879, 189220. 10.1016/j.bbcan.2024.189220 39571764

[bib57] Zhao M., Mishra L., Deng C.X. (2018). The role of TGF-**β**/SMAD4 signaling in cancer. Int. J. Biol. Sci. 14, 111–123. 29483830 10.7150/ijbs.23230 PMC5821033

[bib58] Zhao S., Venkatasubbarao K., Lazor J.W. et al. (2008). Inhibition of STAT3 Tyr705 phosphorylation by Smad4 suppresses transforming growth factor β-mediated invasion and metastasis in pancreatic cancer cells. Cancer Res. 68, 4221–4228. 18519681 10.1158/0008-5472.CAN-07-5123

[bib59] Zhao S., Wang Y., Cao L. et al. (2010). Expression of oncogenic K-ras and loss of Smad4 cooperate to induce the expression of EGFR and to promote invasion of immortalized human pancreas ductal cells. Int. J. Cancer 127, 2076–2087. 20473902 10.1002/ijc.25412 PMC2932752

[bib60] Zhou Y., Zhou B., Pache L. et al. (2019). Metascape provides a biologist-oriented resource for the analysis of systems-level datasets. Nat. Commun. 10, 1523. 30944313 10.1038/s41467-019-09234-6 PMC6447622

