# Supplementary material for: Identification and characterization of a TGF-β-independent SMAD4–NFATc1–STAT3 regulatory axis
Source: J Mol Cell Biol. 2025 Aug 26;17(6):mjaf028. doi: 10.1093/jmcb/mjaf028 (PMC12831459; doi:10.1093/jmcb/mjaf028)
Supplement: mjaf028_Supplemental_File [file mjaf028_supplemental_file.pdf]

Supplementary Figure

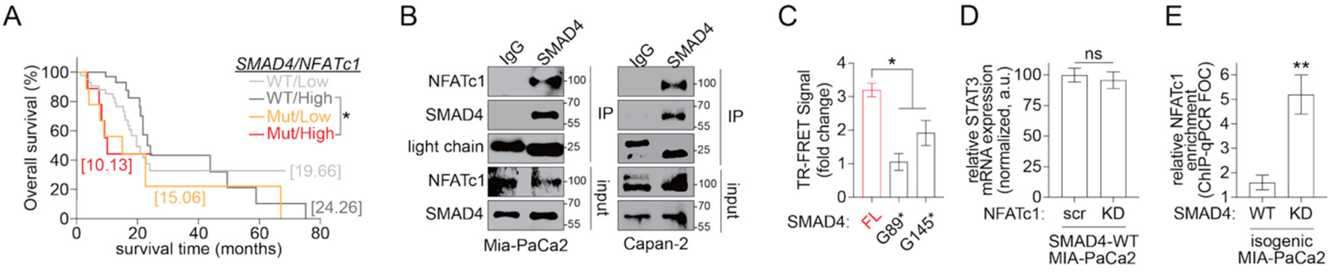

Supplementary Figure 1 (**A**) The Kaplan–Meier curves showing decreased survival in PDAC patients with SMAD4 mutation plus NFATc1 overexpression. NFATc1 low or high expression samples were defined based on NFATc1 mRNA transcript abundance below or above the median in the TCGA-PDAC cohort. **P* < 0.05 by log-rank test. (**B**) Representative western blot images showing endogenous interaction of SMAD4–NFATc1 using co-IP in Mia-PaCa2 (left) and Capan-2 (right) cells. (**C**) Bar graph showing the effect of SMAD4 truncation mutants on SMAD4–NFATc1 PPI signal in TR-FRET assay. Lysates from HEK293T cells co-expressing GST-SMAD4 full-length (FL), G89* truncation mutant, or G145* truncation mutant with Venus-flag-tagged NFATc1 were used in TR-FRET assay with anti-GST-Tb antibody as donor and Venus fluorescence protein as acceptor. Data are presented as mean ± SD from three independent biological replicates; **P* < 0.05 by student's *t*-test. (**D**) Bar graph showing relative STAT3 mRNA expression upon NFATc1 knockdown (KD) in the SMAD4-wild type (WT) MIA-PaCa2 cells. STAT3 mRNA was quantified by qPCR using STAT3-specific primers, as described in Supplementary Methods. Data represent mean ± SD of three independent biological replicates; ns, *P* > 0.05 by student's *t*-test. (**E**) Bar graph showing relative NFATc1 enrichment at the STAT3 promoter region in isogenic MIA-PaCa-2 cells. ChIP was performed using SMAD4-WT and SMAD4-KD cells with an anti-NFATc1 antibody. Enrichment at the STAT3 promoter was quantified by qPCR using STAT3 promoter-specific primers, as described in Supplementary Methods. Data represent mean ± SD of three independent biological replicates; ***P* < 0.01 by student's *t*-test.

Supplementary Methods

BRET-based OncoPPI mapping

PPIs between SMAD4 and 556 cancer-associated proteins were assessed in a miniaturized 1536-well plate-based format in live HEK293T cells. NLuc-tagged SMAD4 and Venus-tagged OncoPPI proteins enabled monitoring of both protein expressions and PPI signals. Binary co-transfections were performed using polyethylenimine (PEI) transfection reagent (Cat# 23966) with automated Biomek NX^P Lab Automation Workstation (Beckman Coulter). BRET PPI signal and relative protein expression signal were measured 48 h after transfection using an Envision Multilabel plate reader (PerkinElmer). PPI signals were analyzed by fitting BRET saturation curves, and the area under the curve (AUC) was calculated to integrate signal amplitude and shape. Fold-of-change (FOC) and statistical significance (P_{FOC}) were determined relative to control samples using CARINA algorism as previously described (Mo et al., 2022).

TR-FRET-based OncoPPI mapping

For the TR-FRET-based OncoPPI mapping, PPIs between SMAD4 and 556 cancer-associated proteins were assessed in a miniaturized 384-well plate-based format in HEK293T cell lysate. GST-tagged SMAD4 and Venus-tagged OncoPPI proteins enabled monitoring of both protein expressions and PPI signals. Binary co-transfections were performed using PEI transfection reagent with automated Biomek NX^P Lab Automation Workstation (Beckman Coulter). Cell lysates were prepared 48 h after transfection by three freeze–thaw cycles in FRET buffer (20 mM Tris-HCl, pH 7.0, 50 mM NaCl, and 0.01% NP-40) containing anti-GST-Terbium antibody (1:1000, Cisbio US Inc., Cat# 61GSTTLB). The plate was centrifuged at 200× *g* for 5 min and incubated at 4°C overnight. TR-FRET signals were measured using the BMG Labtech PHERAstar *FSX* reader with the HTRF optic module (excitation at 337 nm, emission A at 665 nm, emission B at 620 nm, integration start at 50 ms, integration time for 150 ms, and 8 flashes per well) (Xiong et al., 2018). All FRET signals were expressed as a TR-FRET ratio: F_{665nm} /F_{620nm} × 10^4.

Transfection

PEI transfection reagent was used for plasmid transfection in OncoPPI mapping (Li et al., 2017; Mo et al., 2017, 2022). FuGene HD (Roche, Cat# E2920) was used in a ratio of 3 µl to 1 µg DNA for transfection in other experiments.

GST-pulldown assay

Briefly, cell lysates were prepared in 200 µl NP-40 lysis buffer containing 1% NP-40 (IGEPAL CA-630, Sigma-Aldrich), 20 mM Tris-HCl, 150 mM NaCl, 5% glycerol, and 2 mM EDTA, supplemented with protease inhibitor cocktail (Sigma-Aldrich, Cat# P8340), phosphatase inhibitor cocktail 2 (Sigma-Aldrich, Cat# P5726), and phosphatase cocktail 3 (Sigma-Aldrich, Cat# P0044), and subjected to affinity-based pulldown using glutathione-conjugated sepharose beads (GE, Cat# 17-0756-05) for GST-pulldown. Cell lysates were incubated with beads at 4°C for 2 h. Beads were washed with NP-40 lysis buffer for three times. Pulldown or immunoprecipitated protein complexes were eluted by boiling the beads at 95°C for 5 min in 2× Laemmli buffer (Bio-Rad, Cat# 1610737) supplemented with 200 mM DL-dithiothreitol (DTT). Samples were then analyzed by sodium dodecyl sulfate–polyacrylamide gel electrophoresis (SDS–PAGE) and immunoblotting with desired antibodies.

Western blotting (immunoblotting)

Proteins in the SDS sample buffer were resolved by 10% SDS–PAGE and transferred to nitrocellulose filter membranes at 100 V for 2 h at 4°C. After blocked in 5% nonfat dry milk in 1× TBST (20 mM Tris-base, 150 mM NaCl, and 0.05% Tween 20) for 1 h at room temperature, membranes were blotted with the indicated antibodies at 4°C overnight. Afterwards, membranes were washed by 1× TBST for three times, 15 min each time. SuperSignal West Pico PLUS Chemiluminescent Substrate (Thermo, #34580) and Dura Extended Duration Substrate (Thermo, #34076) were used for developing membranes. The luminescence images were captured using ChemiDoc™ Touch Imaging System (Bio-Rad).

Co-immunoprecipitation with endogenous proteins

Briefly, cell lysates were prepared in NP-40 lysis buffer. DTT (10 mM) and N-ethylmaleimide (5 mM) were added into cell lysates used for protein ubiquitination level measurement. Cell lysates with ~1.5 mg of total proteins were used for immunoprecipitation by incubating with desired protein antibody or IgG control at 4°C for 16 h. Afterwards, protein A/D agarose beads were added to the cell lysate and antibody mixture for incubation at 4°C for 1 h. Beads were then washed with NP-40 lysis buffer three times. Immunoprecipitated protein complexes were eluted by IgG elution buffer (Thermo Scientific, Cat# 21028), with DTT added

at a final concentration of 20 mM, and boiled for another 5 min at 95°C. Samples were analyzed by SDS–PAGE and immunoblotting with desired antibodies.

Bimolecular fluorescence complementation assay (BiFC)

Briefly, cells were co-transfected with SMAD4 and NFATc1 conjugated to C-terminal or N-terminal fragments of Venus. The cells transfected with empty vector served as controls. Proteins were expressed for 48 h, and the PPIs were monitored in live cells based on the fluorescence intensity of reconstituted Venus. The cell nuclei were stained with 5 mg/ml Hoechst 33342 (ThermoFisher, H3570) for 30 min at 4°C. The fluorescence intensity of reconstituted Venus was measured on the Envision spectrophotometer (excitation 485 nm and emission 535 nm, mirror 505 nm). The cell images were taken with Olympus FV1000 inverted confocal microscope at 447 nm (Hoechst nuclei stain) and 530 nm (Venus green fluorescence).

Antibodies for western blotting

The following antibodies were used for western blotting: Flag-HRP (Sigma, Cat# A8592, dilution 1:4000), GST-HRP (Sigma, Cat# A7340, dilution 1:5000), GST (Cell Signaling Technology, Cat# 2624S, dilution 1:1000), Flag (Cell Signaling Technology, Cat# 14793, dilution 1:1000), b-actin (Sigma, Cat# A5441, dilution 1:5000), STAT3 (Cell Signaling Technology, Cat# 4904S, dilution 1:1000), phosphor-STAT3 (Y705) (Cell Signaling Technology, Cat# 9145S, dilution 1:1000), NFATc1 (Santa Cruz, Cat# sc-7294, dilution 1:1000), and p-NFATc1(S237) (Abcam, Cat# ab183023, dilution 1:1000).

Generation of lentivirus

HEK293T cells (5 × 10^6) were seeded into a 6-well plate and transfected using PEI transfection reagent with 2 μg of shRNA or sgRNA vectors, together with 1.6 μg pCMV-dR8.91 and 0.66 μg of pCMV-VSVG (Ng et al. 2018). After 48–72 h, the conditioned media containing lentivirus particles were collected and centrifuged at 4°C, 2500× *g* for 15 min. Then, the media were filtered by 0.45-μm PVDF filter (Millipore, Cat# SLHV033RS) and stored at −80°C.

Quantitative real-time polymerase chain reaction (qPCR)

Total RNA was isolated from cell lysates using E.Z.N.A.® Total RNA Kit I (Omega, Cat# R6834-01) and digested with DNase I (Invitrogen, Cat# 18068-015). Approximately 1 μg of RNA was used for cDNA synthesis using SuperScript™ III First-Strand Synthesis System (Invitrogen,

Cat# 18080051) following the manufacture's instruction. Reverse-transcribed cDNA or isolated cytoplasmic DNA was diluted 1:5–1:10 in nuclease-free water. qPCR was performed using SYBR Green Supermix (Bio-Rad, Cat# 1725272) in Mastercycler® RealPlex PCR System (Eppendorf) with STAT3 primers (F: 5'-CTTTGAGACCGAGGTGTATCACC-3'; R: 5'-GGTCAGCATGTTGTACCACAGG-3') ordered from Integrated DNA Technologies. The following thermal cycling conditions were used: 95°C for 2 min followed by 40 thermal cycles of 95°C for 15 sec, 60°C for 15 sec, and 72°C for 20 sec. RNA expression was normalized to GAPDH expression, and relative expression was calculated using the comparative $2^{-\Delta\Delta Ct}$ method.

Chromatin immunoprecipitation followed by qPCR (ChIP–qPCR)

ChIP–qPCR was performed with the SimpleChIP® Plus Sonication Chromatin IP Kit (Cell Signaling Technology, #56383) using the isogenic MIA-PaCa-2 SMAD4-WT and SMAD4-KD cell line. Briefly, cells were formaldehyde-cross-linked (1%, 10 min), lysed, and sonicated with the kit buffers. Equal chromatin aliquots were incubated overnight at 4°C with 5 µg anti-NFATc1 antibody (Santa Cruz, Cat# sc-7294) or normal mouse IgG supplied in the kit, captured with the magnetic Protein A/G beads in the kit, washed, de-cross-linked, and column-purified exactly as instructed. Enrichment of NFATc1 at the STAT3 promoter/enhancer was quantified on a Mastercycler® RealPlex PCR System (Eppendorf) with primer pairs flanking the −674/−661 cluster (F: 5'-TTTCTGCGTGAGCAGGGACACA-3', R: 5'-CCACTACCCTCTCCCCACGCAC-3'). Relative enrichment was calculated using the comparative $2^{-\Delta\Delta Ct}$ method. NFATc1 occupancy was expressed relative to the corresponding SMAD4-WT signal.

Proteomics sample preparation

Cells were washed in phosphate-buffered saline and frozen before proteomics analysis. Frozen cells were resuspended in 100 mM Tris (pH 8) containing 1% SDC, 10 mM TCEP, and 40 mM CAA. The samples were boiled at 95°C for 10 min and then sonicated using a Bioruptor (Diagenode) for 10 cycles of 30 sec on and 30 sec off. Protein concentration was measured using a BCA assay (Thermo Fisher Scientific). Proteins were digested overnight at 37°C with Trypsin/Lys-C Mix (1:100 *w*/*w*; Promega). Peptides were cleaned up on StageTips (Thermo Fisher Scientific), quantified using a Quantitative Colorimetric Peptide Assay (Thermo Fisher Scientific) according to the manufacturer's instructions and dried in a SpeedVac.

LC–MS/MS

Samples were resuspended in buffer A (0.1% FA in water) and 200 ng per sample was injected into a Bruker NanoElute coupled to a timsTOF Pro2 mass spectrometer (Bruker Daltonics). The peptides were loaded on a 25-cm Aurora Elite CSI column (IonOpticks). The mass spectrometer operated in positive polarity for data collection using data-independent acquisition (diaPASEF) mode (Meier et al., 2020). DIA isolation windows were optimized by py_diAID, consisting of 2 ion mobility windows and 25 variable mass isolation windows (Skowronek et al., 2022). All spectra were acquired within an m/z range of 100–1700 and fragmentation energy was set to 20 eV at 0.85 1/K0 and 59 eV at 1.30 1/K0.

Proteomics data analysis

Protein identification and quantification were performed using Spectronaut (Bruderer et al., 2017) (version 17) in library-free mode using the Homo sapiens SwissProt database (20375 entries) with default settings. Modifications were defined as follows: Carbamidomethylation (C) as a fixed modification and Acetyl (Protein N-term) and Oxidation (M) as variable modifications. All further data processing was performed in R. Intensities were normalized by median centering and $\log_2$-scaling. The missing values were imputed by k-nearest neighbors (k-NN) imputation and downshift sampling, for proteins missing at random or not at random, respectively (Lazar et al., 2016). All statistical comparisons between two groups were performed based on two-tailed Student's *t*-tests. Data are available via ProteomeXchange with identifier PXD067749.

Supplementary References

Bruderer, R., Bernhardt, O.M., Gandhi, T., et al. (2017). Optimization of experimental parameters in data-independent mass spectrometry significantly increases depth and reproducibility of results. Mol Cell Proteomics *16*, 2296-2309.

Lazar, C., Gatto, L., Ferro, M., et al. (2016). Accounting for the multiple natures of missing values in label-free quantitative proteomics data sets to compare imputation strategies. J Proteome Res *15*, 1116-1125.

Li, Z., Ivanov, A.A., Su, R., et al. (2017). The OncoPPi network of cancer-focused protein–protein interactions to inform biological insights and therapeutic strategies. Nat Commun *8*, 14356.

Meier, F., Brunner, A.D., Frank, M., et al. (2020). diaPASEF: parallel accumulation-serial fragmentation combined with data-independent acquisition. Nat Methods *17*, 1229-1236.

Mo, X., Niu, Q., Ivanov, A.A., et al. (2022). Systematic discovery of mutation-directed neo-protein–protein interactions in cancer. Cell *185*, 1974-1985.e12.

Mo, X., Qi, Q., Ivanov, A.A., et al. (2017). AKT1, LKB1, and YAP1 revealed as MYC interactors with NanoLuc-based protein-fragment complementation assay. Mol Pharmacol *91*, 339-347.

Ng, P.K., Li, J., Jeong, K.J., et al. (2018). Systematic functional annotation of somatic mutations in cancer. Cancer Cell *33*, 450-462.e10.

Xiong, J., Pecchi, V.G., Qui, M., et al. (2018). Development of a time-resolved fluorescence resonance energy transfer ultrahigh-throughput screening assay for targeting the NSD3 and MYC interaction. Assay Drug Dev Technol *16*, 96-106.

Skowronek, P., Thielert, M., Voytik, E., et al. (2022). Rapid and in-depth coverage of the (phospho-)proteome with deep libraries and optimal window design for dia-PASEF. Mol Cell Proteomics *21*, 100279.
